# Characterization of Small Interfering RNAs Derived from the Geminivirus/Betasatellite Complex Using Deep Sequencing

**DOI:** 10.1371/journal.pone.0016928

**Published:** 2011-02-09

**Authors:** Xiuling Yang, Yu Wang, Wei Guo, Yan Xie, Qi Xie, Longjiang Fan, Xueping Zhou

**Affiliations:** 1 State Key Laboratory of Rice Biology, Institute of Biotechnology, Zhejiang University, Hangzhou, People's Republic of China; 2 Institute of Crop Science, Zhejiang University, Hangzhou, People's Republic of China; 3 State Key Laboratory of Plant Genomics, Institute of Genetics and Developmental Biology, Chinese Academy of Sciences, Beijing, People's Republic of China; University of Wisconsin-Milwaukee, United States of America

## Abstract

**Background:**

Small RNA (sRNA)-guided RNA silencing is a critical antiviral defense mechanism employed by a variety of eukaryotic organisms. Although the induction of RNA silencing by bipartite and monopartite begomoviruses has been described in plants, the nature of begomovirus/betasatellite complexes remains undefined.

**Methodology/Principal Findings:**

*Solanum lycopersicum* plant leaves systemically infected with Tomato yellow leaf curl China virus (TYLCCNV) alone or together with its associated betasatellite (TYLCCNB), and *Nicotiana benthamiana* plant leaves systemically infected with TYLCCNV alone, or together with TYLCCNB or with mutant TYLCCNB were harvested for RNA extraction; sRNA cDNA libraries were then constructed and submitted to Solexa-based deep sequencing. Both sense and anti-sense TYLCCNV and TYLCCNB-derived sRNAs (V-sRNAs and S-sRNAs) accumulated preferentially as 22 nucleotide species in infected *S. lycopersicum* and *N. benthamiana* plants. High resolution mapping of V-sRNAs and S-sRNAs revealed heterogeneous distribution of V-sRNA and S-sRNA sequences across the TYLCCNV and TYLCCNB genomes. In TYLCCNV-infected *S. lycopersicum* or *N. benthamiana* and TYLCCNV and *βC1*-mutant TYLCCNB co-infected *N. benthamiana* plants, the primary TYLCCNV targets were AV2 and the 5′ terminus of AV1. In TYLCCNV and betasatellite-infected plants, the number of V-sRNAs targeting this region decreased and the production of V-sRNAs increased corresponding to the overlapping regions of AC2 and AC3, as well as the 3′ terminal of AC1. *βC1* is the primary determinant mediating symptom induction and also the primary silencing target of the TYLCCNB genome even in its mutated form.

**Conclusions/Significance:**

We report the first high-resolution sRNA map for a monopartite begomovirus and its associated betasatellite using Solexa-based deep sequencing. Our results suggest that viral transcript might act as RDR substrates resulting in dsRNA and secondary siRNA production. In addition, the betasatellite affected the amount of V-sRNAs detected in *S. lycopersicum* and *N. benthamiana* plants.

## Introduction

Since the discovery in 1993 of the first small RNAs (sRNAs) [1], RNA silencing machinery has been described in a wide range of eukaryotic organisms. In plants, the steps associated with the RNA silencing pathway include: (*a*) the formation of double stranded RNA (dsRNA) from internal base-paired stem loop structures, convergent transcription of transposons and transgenes or RNA dependent RNA polymerase (RDRs)-directed synthesis from single stranded RNA (ssRNA); (*b*) recognition and cleavage of dsRNA into small interfering RNAs (siRNAs) by RNase III-like enzymes; (*c*) 3′ methylation of siRNA at the 2′-hydroxyl group and (*d*) incorporation of siRNA into effector complexes that guide sequence-specific inactivation of target mRNAs or DNAs [2]. *Arabidopsis thaliana* represents the best-defined plant model for the characterization of RNA silencing machinery. This plant possesses four Dicer-like proteins (DCLs), five dsRNA-binding proteins (DRBs), six RDRs and ten Argonaute proteins (AGOs) [3]. These core silencing factors constitute at least four distinct sRNA-directed silencing pathways, including micro-RNA (miRNA)-directed gene regulation, *trans*-acting siRNAs (*ta*-siRNAs) that mediate negative gene regulation, natural antisense transcript-associated siRNAs (nat-siRNAs) involved in plant stress responses and heterochromatic siRNAs that direct DNA and histone methylation [4]. The first three pathways involve post-transcriptional gene silencing (PTGS)-related processes in which DCL1 and AGO1 are required predominantly for the biogenesis of the 21 nucleotide (nt) species of micro-RNAs; DCL2, RDR6 and DCL4 function in nat-siRNAs and *ta*-siRNAs pathways, respectively. Heterochromatic siRNA-directed epigenetic phenomena involve transcriptional gene silencing (TGS)-related process which require RDR2, DCL3 and AGO4 or AGO6 for the biogenesis of the 24 nt siRNAs [2], [5].

The family of *Geminiviridae* is one of the few plant virus families that have single stranded DNA genomes and these viruses have caused significant economic losses due to their capacity to destroy a wide range of plants. These viruses replicate in the nucleus by a rolling circle mechanism via a double-stranded DNA (dsDNA) replicative form (RF) intermediates that associate with histones to form minichromosomes [6]–[9]. Four genera have been classified from hundreds of known geminiviruses and the *Begomovirus* is the largest genus with more than 200 species [10]. Most members of this genus have a bipartite genome designated as DNA-A and DNA-B which are similar in size (about 2.7 kb) and usually necessary to establish systemic infections. Some begomoviruses have only a single genome that is equivalent to the DNA-A component of the bipartite begomoviruses containing all of the genetic information necessary for systemic infections and the induction of characteristic symptoms. Many monopartite begomoviruses are associated with a betasatellite which is about 1.3 kb in size and indispensable for the induction of infection by some monopartite begomoviruses [11]–[13].

Like most RNA viruses and some viroids, DNA geminiviruses are both inducers and targets of RNA silencing. Following geminivirus infections, three distinct classes of virus-derived siRNAs (V-sRNA) are found in infected host cells [14]–[16]. Geminiviruses encode diverse RNA silencing suppressors that block one or more distinct steps of the RNA silencing pathway [17]–[21]. However, several aspects concerning the biogenesis and origin of geminivirus-derived siRNAs in plants remain obscure.

Tomato yellow leaf curl China virus (TYLCCNV) is a monopartite begomovirus associated with a betasatellite (TYLCCNB). TYLCCNV can systemically infect *Nicotiana benthamiana*, *N. glutinosa*, *Petunia hybrida* and *Solanum lycopersicum* plants, but only induces typical leaf curl symptoms in the presence of the betasatellite. Mutation of the betasatellite *βC1* gene abolished its ability to induce typical symptoms and reduced infectivity [22]. In this study, we analyzed the composition and the molecular nature of TYLCCNV- and TYLCCNB-derived siRNAs (V-sRNAs and S-sRNAs, respectively) through deep sequencing. We report the first high-resolution sRNA map for a monopartite begomovirus and its associated betasatellite.

## Results

### Identification of V-sRNA and S-sRNA populations

**Figure 1:**
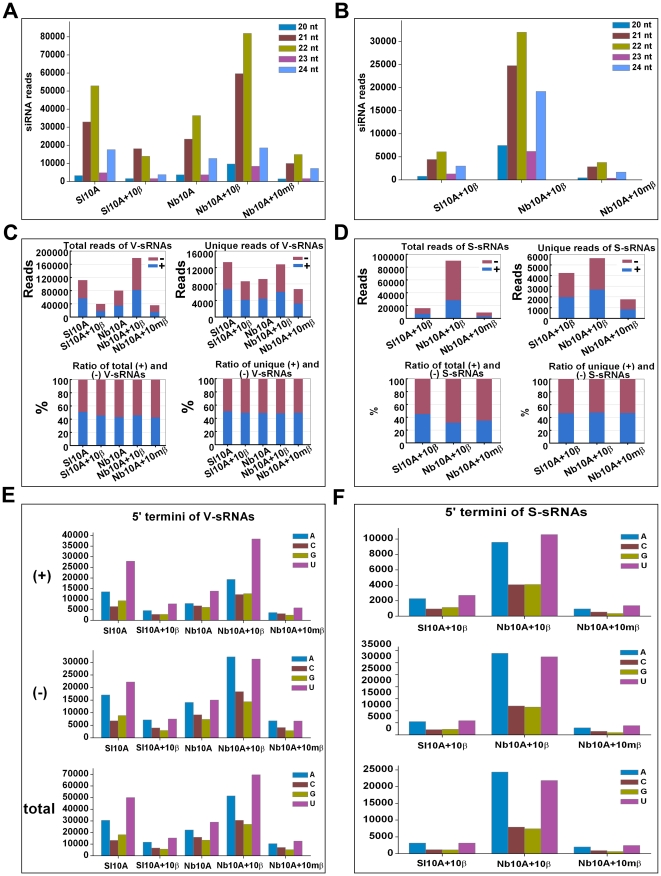
Identification of TYLCCNV-derived sRNAs (V-sRNAs) and TYLCCNB-derived sRNAs (S-sRNAs) obtained from virus infected *S. lycopersicum* and *N. benthamiana* plants. (**A, B**) The size distribution of cloned 20 to 24 nt V-sRNAs and S-sRNAs. (**C, D**) Polarity distribution of cloned V-sRNAs and S-sRNAs, respectively. Histograms represent the reads and ratios of total or unique V-sRNA (**C**) or S-sRNA (**D**) within each category. (**E, F**) The relative abundance of the four different 5′-terminal nucleotides of V-sRNA and S-sRNA, respectively. The values for each distinct 5′-terminal nucleotide were calculated according to total V-sRNA or S-sRNA reads. Only reads that were 100% identical or complementary to the TYLCCNV or TYLCCNB genomes were included in the analysis. Sl10A and Sl10A+10β represent *S. lycopersicum* plants infected with TYLCCNV alone or in combination with TYLCCNB, respectively; Nb10A, Nb10A+10β and Nb10A+10mβ represent *N. benthamiana* plants infected with TYLCCNV alone or in combination with TYLCCNB or *βC1*-mutant TYLCCNB, respectively.

To investigate the composition of V-sRNA and S-sRNA populations, a computational blast was carried out to search for sRNA sequences identical or complementary to sequences present in the TYLCCNV and TYLCCNB genomes. Our results showed that 21, 22 and 24 nt reads predominated the V-sRNA and S-sRNA sequences examined (Figure 1A and 1B), indicating the involvement of multiple DCLs in the biogenesis of V-sRNA and S-sRNA. These observations were consistent with a previous report describing viral sRNA blot-based analysis of Cabbage leaf curl virus (CaLCuV)-derived siRNA that accumulated in *Arabidopsis* RNA silencing mutants [15]. In TYLCCNV-infected *S. lycopersicum* and *N. benthamiana* plants, 22 nt V-sRNAs accumulated to higher levels than the 21 nt species (47.44% vs. 29.49% for *S. lycopersicum* and 45.48% vs. 29.24% for *N. benthamiana*) (Figure 1A). The 22 nt sRNAs peak was also observed for V-sRNA and S-sRNA from TYLCCNV and TYLCCNB co-infected *N. benthamiana* plants but the 21 nt V-sRNAs occupied the dominant peak (46.32%) in TYLCCNV and TYLCCNB co-infected *S. lycopersicum* leaves (Figure 1A and 1B). Our deep sequencing data is somewhat different from previous data obtained using mixed pyrosequencing of Tomato yellow leaf curl virus (TYLCV)-derived sRNAs, showing that among the 1,212 TYLCV-derived sRNAs, the 21 nt species occurred more frequently than the 22 nt species [23]. Given the functionality of 22 nt siRNAs in triggering secondary siRNA biogenesis [24], our results indicated that this might be a defense strategy used by plants against geminivirus infections.

When the polarity of V-sRNAs was evaluated using total V-sRNA reads of 20–24 nt, an approximately equal ratio of sense (+) and antisense (-) V-sRNAs was observed in *S. lycopersicum* and *N. benthamiana* plants infected with either TYLCCNV alone or with TYLCCNB (Figure 1C). A more specific analysis of V-sRNA polarity using reads from each individual size class or using unique sequences showed an essentially similar ratio of sense (+) and antisense (-) V-sRNAs (Figure 1C and Figure S1), indicating that the siRNAs originated from dsRNA. Similar results were observed for the (+) and (-) polarities of S-sRNAs, the only exception being a moderate excess of (-) S-sRNAs (68.49%) in TYLCCNV and TYLCCNB co-infected *N. benthamiana* plants (Figure 1D), suggesting that additional factors different between infected *S. lycopersicum* and *N. benthamiana* plants were involved in S-sRNAs biogenesis.

It has been reported that sorting of sRNA into specific AGO complexes is largely directed by their 5′ terminal nucleotide in *Arabidopsis* and rice [25]–[28]. In order to infer potential interactions with distinct AGO complexes, the nucleotide at the 5′ terminal position was analyzed in V-sRNAs and S-sRNAs sequences, respectively. Bioinformatic analysis revealed preferential use of uridine (U) and adenosine (A) residues compared to cytosine (C) and guanidine (G) for both V-sRNAs and S-sRNAs, regardless of the inoculums or the host tested (Figures 1E and 1F). Detailed analysis of total V-sRNA or S-sRNA reads of 21, 22, or 24 nt in length showed an intriguing bias: for both V-sRNAs and S-sRNAs, 21 and 22 nt in length, both (+) and (-) polarity species presented with a dominant or co-dominant U at their 5′-terminal position, while the 24 nt V-sRNA species presented with a dominant A in most cases except for (+) V-sRNAs captured from *S. lycopersicum* plants infected with TYLCCNV alone or together with TYLCCNB (Figure S2). In *Arabidopsis*, AGO2 and AGO4 preferentially recruited sRNAs with a 5′ terminal A, whereas AGO1 predominantly favored a 5′ terminal U [25]–[27]. Our results suggested that V-sRNAs and S-sRNAs might be loaded into diverse AGO-containing silencing complexes.

### Hotspots for generation of sRNA in the TYLCCNV and TYLCCNB genomes

**Figure 2:**
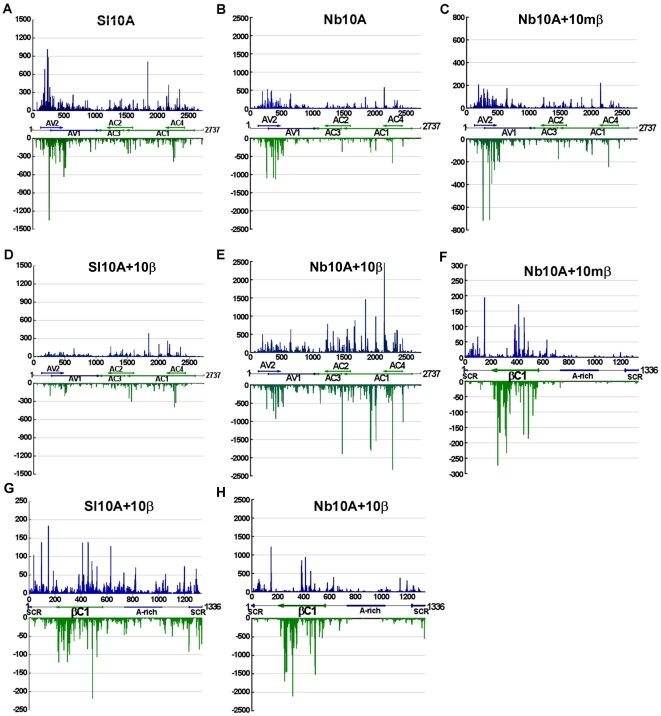
Genome views of V-sRNAs and S-sRNAs obtained from virus infected *S. lycopersicum* and *N. benthamiana* plants. (**A, B**) V-sRNAs sequences captured from TYLCCNV-infected *S. lycopersicum* and *N. benthamiana* plants, respectively; (**D,G,E,H**) V-sRNAs and S-sRNAs sequences captured from TYLCCNV and TYLCCNB co-infected *S. lycopersicum* and *N. benthamiana* plants, respectively; (**C, F**) V-sRNAs and S-sRNAs sequences captured from TYLCCNV and *βC1*-mutated TYLCCNB co-infected *N. benthamiana* plants, respectively. The values were calculated according to total reads of V-sRNAs and S-sRNAs. Positive values corresponded to sense sRNAs and negative values corresponded to antisense sRNAs. Genome organization of TYLCCNV and TYLCCNB are indicated in linear form. The open reading frames encoded on the viral and complementary strand are shown, respectively. The accession numbers of the reference sequence for TYLCCNV and TYLCCNB are AJ319675 and AJ421621, respectively. Representation of Sl10A, Sl10A+10β, Nb10A, Nb10A+10β or Nb10A+10mβ was the same as in Figure 1.

To examine the genomic distribution of V-sRNAs, the 5′-terminus of V-sRNAs captured from TYLCCNV-infected *S. lycopersicum* and *N. benthamiana* plants were plotted against the TYLCCNV genome according to their polarities and genomic locations. Several distribution patterns were observed in TYLCCNV-infected *S. lycopersicum* and *N. benthamiana* plants (Figures 2A and 2B). Firstly, V-sRNAs nearly saturated the viral genome including the coding and intergenic regions. Secondly, V-sRNAs originating from the sense and antisense strands were distributed equally. Thirdly, both sense and antisense V-sRNAs displayed a strong non-uniform distribution pattern along the genome with a large proportion of V-sRNAs concentrating in specific regions. Further estimation of the V-sRNA-generating hotspots in TYLCCNV showed that the region corresponding to AV2 and the 5′ terminal region of AV1 (covering only about 15% of the TYLCCNV genome) had a tendency to contain higher levels of V-sRNAs with more than 40% of the sequenced V-sRNAs clustered in this region (Figures 2A and 2B). In contrast, the intergenic region had a tendency to accumulate lower levels of V-sRNAs with only about 2% of the V-sRNAs located in this region (Figures 2A and 2B), indicating that the TYLCCNV genome contains regions that serve as preferential targets of V-sRNA production. Fourthly, the most prominent peaks of sequence abundance corresponding to 21 nt V-sRNAs usually localized to the same genomic regions as peaks corresponding to 22 or 24 nt V-sRNAs (Figure S3), indicating that DCLs have a similar targeting preference toward the same regions along the viral genome.

Since TYLCCNB enhanced viral DNA accumulation and induced typical leaf curl symptoms in infected plants [22], distribution of V-sRNAs was compared between TYLCCNV-infected and TYLCCNV/TYLCCNB co-infected *S. lycopersicum* and *N. benthamiana* plants (Figures 2D and 2E). In contrast to the strong bias for AV2 and the 5′ terminal region of AV1 V-sRNAs in TYLCCNV-infected plants, V-sRNAs corresponding to this region were not more abundant in TYLCCNV/TYLCCNB co-infected *S. lycopersicum* and *N. benthamiana* plants. Instead, a modest increase in the production of V-sRNAs corresponding to AC2, AC3 or the 3′ terminus of AC1 was observed. This indicated that the massive production of V-sRNAs targeting AV2 and the 5′ terminal region of AV1 were inhibited by the presence of TYLCCNB.

Analysis of the V-sRNAs in TYLCCNV and *βC1*-mutated TYCCNB co-infected *N. benthamiana* plants showed that more than 50% of the sequenced V-sRNAs clustered in the region corresponding to AV2 and the 5′ terminal region of AV1, displaying a similar distribution pattern to those sequenced from TYLCCNV-infected *N. benthamiana* plants (Figure 2C). These results indicated that betasatellite-encoded βC1 could alter the V-sRNA-generating hotspots.

The distribution of S-sRNAs was also analyzed. A high proportion of S-sRNAs was derived from the only ORF of TYLCCNB in TYLCCNV/TYLCCNB co-infected *S. lycopersicum* (44.5%) and *N. benthamiana* plants (68.9%) (Figures 2G and 2H). A similar βC1 ORF preference was also found in TYLCCNV and *βC1*-mutant TYLCCNB co-infected *N. benthamiana* plants (79.4%) (Figure 2F and Figure S4).

### Confirmation of sRNA-generating hotspots using reverse Northern blot analysis

**Figure 3:**
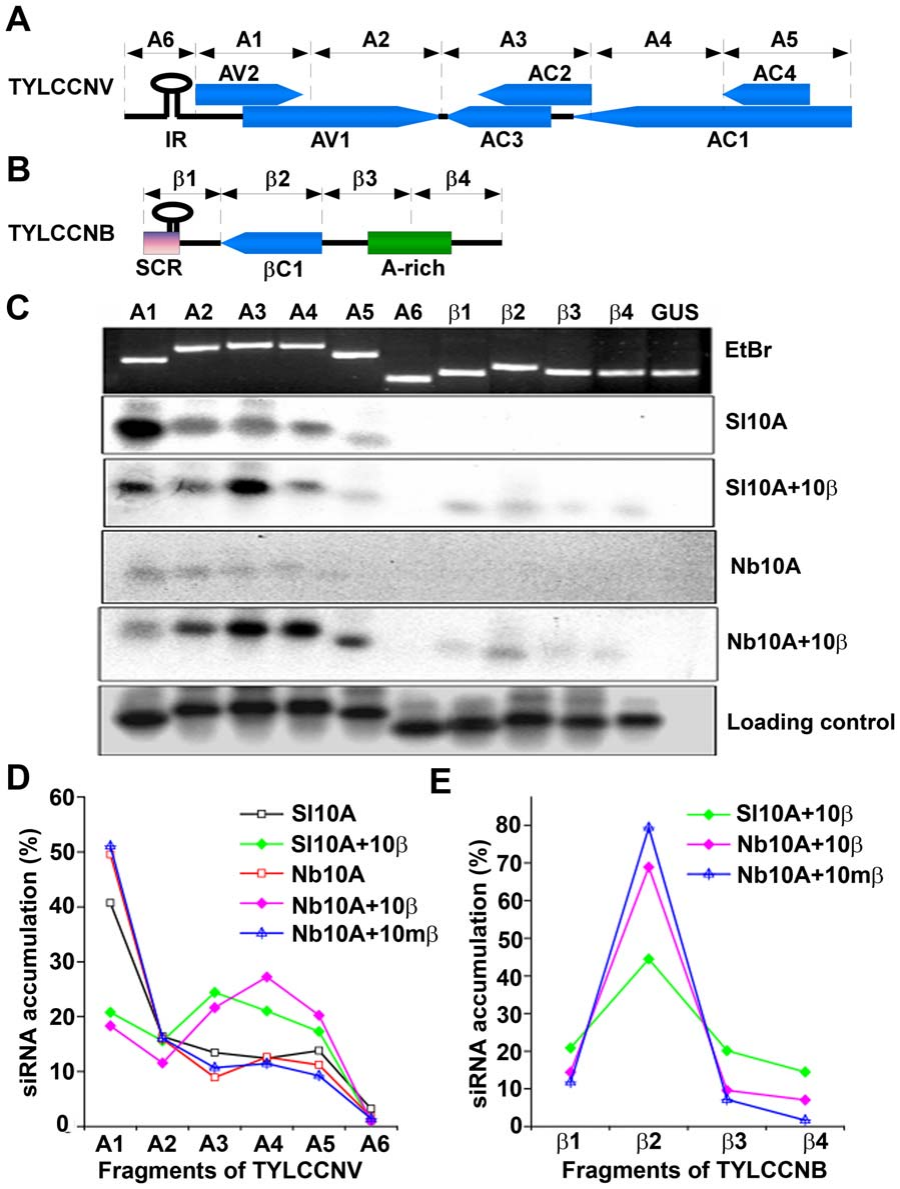
Relative abundance of sRNAs targeting different regions of the TYLCCNV and TYLCCNB genomes, respectively. (**A, B**) Genome organization of TYLCCNV and TYLCCNB, respectively, schematically depicted in linear forms. (**C**) Reverse Northern blot analysis of V-sRNA and S-sRNA. PCR-amplified fragments covering the TYLCCNV and TYLCCNB genomes were obtained and probed with 5′-radiolabeled sRNAs purified from inoculated *S. lycopersicum* or *N. benthamiana* plants. A 320 base pair DNA fragment corresponding to a partial fragment of the β-glucoronidase gene (GUS) was used as a negative hybridization control. Equal loading (500 ng for each lane) was shown by staining agarose gels with ethidium bromide and by probing the blot with full-length DNA fragments of TYLCCNV and TYLCCNB. (**D, E**) Histograms represent the percentage of sRNAs targeting each DNA fragment analyzed according to the deep sequencing data. Representation of Sl10A, Sl10A+10β, Nb10A, Nb10A+10β or Nb10A+10mβ was the same as in Figure 1.

To verify the observed V-sRNA and S-sRNA distribution patterns, six DNA fragments spanning the entire TYLCCNV genome and four covering the TYLCCNB genome were amplified by PCR using appropriate sequence-specific primers (Figures 3A and 3B). Equal amounts of each DNA fragment were blotted and probed with ^32^P-labeled sRNA molecules purified from infected *S. lycopersicum* and *N. benthamiana* plants, respectively. The results showed that sRNAs isolated from TYLCCNV or TYLCCNV/TYLCCNB co-infected *S. lycopersicum* and *N. benthamiana* plants hybridized to each of the DNA fragments with different intensities (Figure 3C). Extremely low density of V-sRNAs was found targeting the intergenic region of TYLCCNV (Figures 3C and 3D, fragment A6). The 5′ terminal TYLCCNV fragment (fragment A1) produced the strongest signal among the six TYLCCNV fragments tested when blots were probed with sRNAs purified from TYLCCNV-infected *S. lycopersicum* or *N. benthamiana* plants, suggesting that a large proportion of V-sRNAs targeted the AV2 and the 5′ terminal region of AV1 (Figure 3C). Interestingly, when the blotted membrane was probed with sRNAs purified from TYLCCNV/TYLCCNB co-infected *S. lycopersicum* and *N. benthamiana* plants, V-sRNAs corresponding to fragment A1 were no longer more abundant than V-sRNAs corresponding to other fragments. By contrast, fragments A3, A4 and β2 seemed to bind more siRNAs than other TYLCCNV and TYLCCNB fragments since they produced the strongest hybridization signals (Figure 3C). As expected, strong hybridization signals for all viral fragments were observed when the same blot was probed with either TYLCCNV or TYLCCNB and no signals were detected using labeled sRNAs from mock-inoculated plants (Figure 3C and data not shown). Unfortunately, attempts at probing reverse Northern blots with sRNAs purified from TYLCCNV and the TYLCCNB *βC1*-mutant co-infected *N. benthamiana* plants proved to be unsuccessful due to reduced sensitivity of the hybridization assay compared to deep sequencing, indirectly supporting the observation that fewer sRNAs were obtained from TYLCCNV and TYLCCNB *βC1*-mutant co-infected *N. benthamiana* plants. Our reverse Northern blot analyses fits well with the results obtained from the deep sequencing data (Figure 3), demonstrating that the betasatellite inhibits the production of V-sRNAs by targeting the AV2 and 5′ terminal region of AV1.

### Potential precursor for V-sRNA biogenesis

**Figure 4:**
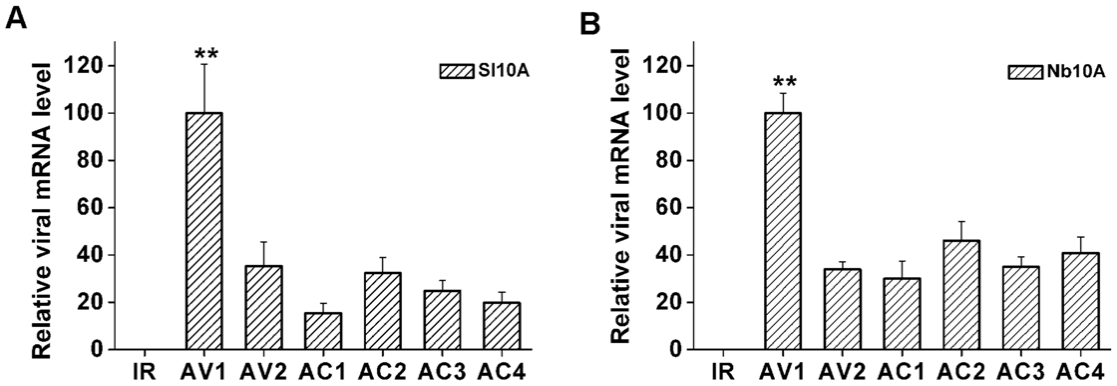
Relative quantification of viral transcripts by quantitative real-time PCR. (**A**) Viral transcript levels from TYLCCNV infected *S. lycopersicum* plants. (**B**) Viral transcript levels from TYLCCNV infected *N. benthamiana* plants. Each bar represents the mean standard error of triplicate readings and the values were calculated using comparative *C*_T_ method. Student's *t*-test was performed to determine whether the higher expression of AV1 was statistically different from the expression of other ORFs. ** represents the expression of AV1 is significantly higher than other ORFs at the 0.01 level (*P*<0.01).

Previous studies have proposed that strong fold-back structures within single-stranded viral transcripts, overlapping transcripts in opposite orientations at the 3′ ends, and abundant aberrant transcripts processed by host RDRs serve as major source of geminivirus dsRNAs that mediate PTGS [29], [30]. We tried to find potential secondary structures that accounted for sRNA-generated hotspots, however, a good correlation between hotspot and potential stem-loop structures predicted by RNAfold (http://rna.tbi.univie.ac.at/) could not be identified (data not shown). To determine whether a heterogeneous distribution of V-sRNAs resulted from different transcription levels of viral proteins, quantitative RT-PCR was performed using total RNA from systemically infected or mock-inoculated *S. lycopersicum* and *N. benthamiana* leaves as template. RNA samples were digested with DNaseI and visualized on agarose gels to confirm the presence of intact RNA templates without DNA contamination. The results showed that, in TYLCCNV-infected *S. lycopersicum* and *N. benthamiana* plants, the mRNA level of AV1 was significantly higher than other ORFs (*t*-test, *P*<0.01), and transcript level of intergenic region was barely detected (Figure 4).

**Figure 5:**
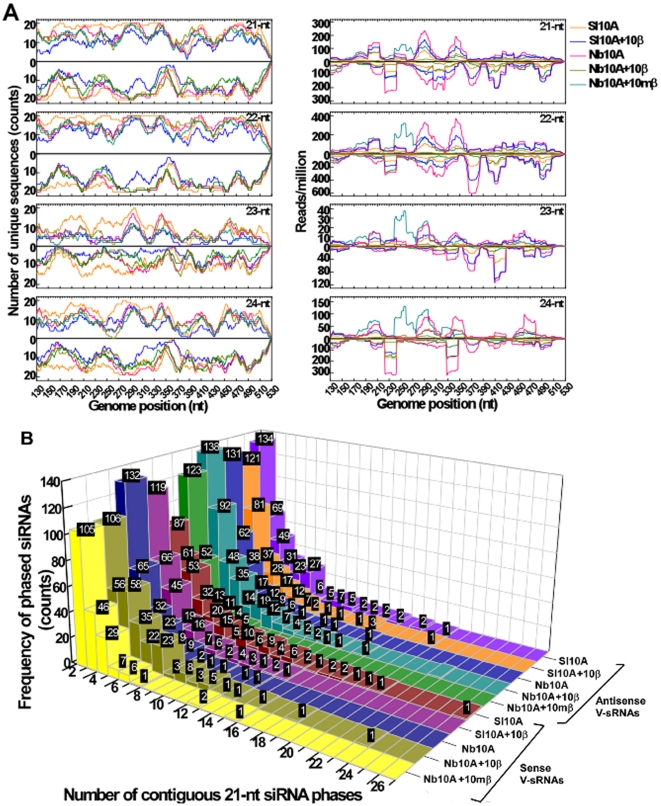
V-sRNA originates from dsRNA. (**A**) A close view of 400 nt TYLCCNV segments showing the sequence complexity and normalized abundance of TYLCCNV V-sRNAs. The left panel shows the number of unique V-sRNAs within each size class plotted against each single-nucleotide sliding window along the viral genome. The right panel shows the abundance of mapped V-sRNAs. The values on the y-axis represent V-sRNAs obtained from the sense strand (above the x-axis) and the antisense strand (below the x-axis), respectively. Data from different sRNA cDNA libraries is indicated in different colors. (**B**) Phased 21 nt V-sRNAs obtained from each sRNA cDNA library. Sense and antisense V-sRNAs from each library are shown separately. The numbers within each column represent the frequencies for phased V-sRNAs with a defined number of successive phases. Representation of Sl10A, Sl10A+10β, Nb10A, Nb10A+10β or Nb10A+10mβ was same as in Figure 1.

To gain further insights into V-sRNA biogenesis, the sequence complexity and normalized abundance of the V-sRNA-generated hotspot was measured as described [5], [31]. The sequence complexity was estimated by calculating the number of unique V-sRNA sequences plotted at each nucleotide position. As shown in the representative 400 nt viral genomic segment, the highest overall sequence complexity of V-sRNA was observed for the 22 nt size class, followed by the 21- and 24 nt size classes, whereas the 23 nt size represented the lowest sequence complexity (Figure 5A). Using a sliding window of 21 or 22, we observed that maximum or near maximum sequence complexity was found for both the sense and antisense V-sRNAs at multiple genomic regions (Figure 5A). In addition, multiple sense/antisense sRNA duplexes with two 3′-protruding nucleotides were found at consecutive positions along the viral genome (data not shown), indicative of DCL-mediated processing of perfectly base-paired, relatively long dsRNAs. These results suggested that these V-sRNAs might derive from a dsRNA precursor that may be processed by DCL2, DCL4 and DCL3 in phases. Searching for phased 21 nt V-sRNAs in each sRNA library showed that up to 26 and 19 contiguous 21 nt phases were found for sense and antisense V-sRNAs, respectively (Figure 5B). Since RDR6 functions in the production of phased *ta*-siRNAs from a cleaved RNA template [32], [33], it is possible that RDR6 may function in V-sRNA biogenesis in a mode similar to *ta*-siRNA biogenesis.

### Effect of betasatellite on the amount of V-sRNAs in *S. lycopersicum* and *N. benthamiana*

**Figure 6:**
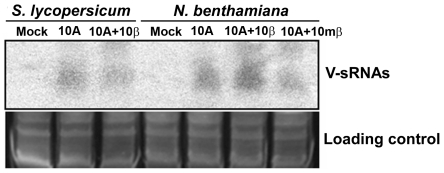
Northern blot analysis of V-sRNAs in different host plants. Low molecular weight (LMW) RNAs were extracted from systemically infected *S. lycopersicum* or *N. benthamiana* plants at 21 dpi. Equal loading of individual LMV RNA samples (40 µg for each lane) is shown by staining of the 15% PAGE gel with SYBR-gold and blot was probed with TYLCCNV full-length DNA fragments. LMV RNA samples isolated from mock-inoculated plants were used as a negative control. 10A represents TYLCCNV, 10β represents TYLCCNB, 10mβ represents the *βC1*-mutant TYLCCNB.

Previous studies have shown that TYLCCNB enhanced viral DNA accumulation in infected plants, and βC1, the unique protein encoded by TYLCCNB, functioned as a suppressor of RNA silencing [17], [22]. To decipher the effect of TYLCCNB on the biogenesis of V-sRNAs, we first compared the amounts of V-sRNAs obtained from sequenced *S. lycopersicum* libraries. Interestingly, the ratio of V-sRNAs dropped from 1.48% in TYLCCNV-infected *S. lycopersicum* plants to 0.5% in TYLCCNV/TYLCCNB co-infected *S. lycopersicum* plants (Table 1), indicating that the betasatellite could inhibit the biogenensis of V-sRNA. Conversely, in sequenced *N. benthamiana* libraries, the presence of the intact betasatellite promoted the production of V-sRNAs from 1.81% to 3.37%, whereas the presence of a *βC1*-mutated betasatellite diminished the production of V-sRNAs to 0.65%. Northern blot analysis of V-sRNAs using the full length TYLCCNV fragment generated a similar result (Figure 6), indicating that interaction between TYLCCNV and TYLCCNB is diverse in different host plants.

**Table 1:** Viruses and host plants used to construct sRNA libraries from virus-infected plants.

Inoculum	Host	sRNA reads	V-sRNA^a^(total reads/percentage)	S-sRNA b(total reads/percentage)
10A^d^	*S. lycopersicum*	7,565,514	111,667/1.48%	N/A^c^
10A+10β^e^	*S. lycopersicum*	7,771,071	391,48/0.50%	15,628/0.20%
10A	*N. benthamiana*	4,430,390	80,121/1.81%	N/A
10A+10β	*N. benthamiana*	5,294,571	178,367/3.37%	8,9765/1.7%
10A+mβ^f^	*N. benthamiana*	5,408,405	35,238/0.65%	9,064/0.17%

a V-sRNA represents TYLCCNV-derived sRNAs.

b S-sRNA represents TYLCCNB-derived sRNAs.

c NA, not applicable.

d 10A represents TYLCCNV.

e 10β represents TYLCCNB.

f 10mβ represents the *βC1*-mutant TYLCCNB.

## Discussion

sRNA-guided RNA silencing functions as an ancient antiviral defense mechanism in a variety of eukaryotic organisms. Virus infection triggers plant PTGS resulting in the production of V-sRNAs in infected plant cells. Recently-developed, next-generation sequencing approaches and computational methods have provided the technology that has allowed investigators to identify an increasing number of small noncoding RNAs and count reads as a quantitative indicator of sequence abundance. In this study, a Solexa-based deep-sequencing approach was used to profile V-sRNA and S-sRNA populations from infected *S. lycopersicum* and *N. benthamiana* plants and we describe the first high-resolution sRNA map from a monopartite begomovirus/betasatellite complex.

Sequence analysis of the deep sequencing data revealed that although production of both V-sRNAs and S-sRNAs were triggered following virus infection, they accounted for a relatively lower proportion of the total reads when compared to levels induced following RNA virus infections [5], [34]. In fact, this variation was also observed following mixed pyrosequencing of nine distinct RNA and DNA viruses [23]. We presumed that this is likely due to the replication strategy used by geminiviruses where replication occurs in the nucleus of infected plant cells, making them less vulnerable to RNA silencing. This may help to explain why current attempts to engineer robust geminiviruse-resistant plants have not been so successful although these strategies have been widely used against RNA virus infection. Recently, Aragao and Faria used intron-spliced hairpin constructs to generate the first transgenic geminivirus-resistant plant [35], however, this achievement was accomplished later than the development of similar RNA viruses-resistant plants [36].

Previous studies have shown that 5′-terminal sRNA nucleotides direct their specific sorting into distinct AGO complexes [25]–[28], [36]. The majority of the 21- and 22 nt V-sRNAs and S-sRNAs recovered in our sequencing pool showed a strong bias for sequences beginning with a 5′-U, indicative of their association with AGO1. This is consistent with the dominant role described for AGO1 in defending against RNA viruses [37]–[40]. Most of the 24 nt V-sRNAs and S-sRNAs displayed a tendency to begin with a 5′-A, suggesting DCL3-mediated biogenesis and association with AGO4. AGO4 is required for the biogenesis and function of heterochromatin-associated siRNAs and is also involved in DNA and chromatin modifications [41]. This suggested that these V-sRNAs and S-sRNAs might associate with AGO4 to direct DNA methylation and TGS at specific genomic loci that share sequence complementarity with V-sRNA and S-sRNA sequences. Indeed, AGO4 was reported to play important role in DNA methylation against geminivirus genome [42].

Analysis of V-sRNA accumulation using *Arabidopsis dcl* double or triple mutants revealed a primary role for DCL4 and a redundant role for DCL2 in antiviral immunity. In contrast with the hierarchical activity of DCL4, DCL2 and DCL3 in the biogenesis of V-sRNAs [23], [43], V-sRNAs and S-sRNAs of 22 nt were the most highly represented species observed in most of the sequence pools analyzed, regardless of total sRNA reads or unique sRNA sequences. Since DCL2 is known to promote a full silencing response against several viruses and to generate 22 nt siRNAs [15], [43]–[45], one possible explanation is that 22 nt V-sRNAs and S-sRNAs are a consequence of secondary siRNA production. This could be a strategy used by plants to amplify sRNA as a means of eliciting a more potent defense against geminiviruses since it has been recently shown that a length of 22 nt facilitated the triggering of secondary siRNA production [24], [46], [47]. Indeed, significantly enhanced susceptibility to Beet curly top virus (BCTV) and CaLCuV was observed in *dcl2*-deficient plants [42].

Polarity analysis of the sequenced *S. lycopersicum* and *N. benthamiana* pools showed approximately equal ratios of sense:antisense sRNA strands, indicating V-sRNAs would be produced from dsRNA precursors comprised of sense and antisense strands derived from the viral genome. In addition, multiple sense/antisense sRNA duplexes with two 3′-protruding nucleotides at consecutive positions along the viral genome were suggestive of DCL-mediated processing of perfectly base-paired, relatively long dsRNAs. Since RDR6 is involved in DCL-dependent secondary siRNA biogenesis, further comparison of V-sRNA and S-sRNA profiles in geminivirus-infected *rdr* mutant plants will help us to address this question.

Deep sequencing data and reverse northern blot analysis showed variations in the relative abundance of siRNAs targeting different regions of the TYLCCNV and TYLCCNB genomes. Failure to find significant correlations between hotspots and regions predicted to adopt a potential hairpin structure might exclude the possibility that the bulk of V-sRNAs and S-sRNAs originated from direct processing of folded ssRNA. Quantitative RT-PCR revealed that higher transcription levels of the RNA might give rise to an increase in aberrant RDR RNA substrates because the highest levels of viral transcripts were detected at the AV1 region that was preferentially targeted by RNA silencing. In contrast, mRNA transcription in the intergenic region was detected at low levels and few V-sRNAs were derived from the intergenic region. Muangsan *et al.* [48] showed that geminivirus-induced gene silencing was compromised in *Arabidopsis rdr* mutants, demonstrating the importance of RDR6 in efficient targeting against geminiviruses. Hence, due to the deficiency of active RDR1 in *N. benthamiana* plants [49], we speculated that RDR6 was required for the biogenesis of geminivirus-derived sRNAs. However, in TYLCCNV/TYLCCNB co-infected *S. lycopersicum* and *N. benthamiana* plants, the highest transcription levels were also detected in the regions corresponding to AV1 where V-sRNA production was inhibited (data not shown). Therefore, an unanswered question is how betasatellites can alter the generation of V-sRNA hotspots. In a previous study, Wang *et al.* [34] demonstrated that hotspots changing between wild-type, *rdr1* and *rdr1 rdr2 rdr6* plants were due to RDR1 and RDR6 specificity that affected amplification of viral siRNAs from the three genomic RNAs of the Cucumber mosaic virus (CMV). Additional studies will be carried out to determine whether RDR1, RDR6 or other antiviral RDRs preferentially amplify distinct viral genome regions of TYLCCNV in a similar manner to CMV or whether betasatellites target RDR6 or other factors required for RDR6-mediated activity.

According to the results obtained by quantitative RT-PCR and deep sequencing, it is reasonable to infer that the viral mRNAs were targeted by V-sRNAs. This does not mean, however, that V-sRNAs generated from hotspots were more efficient than other V-sRNAs since recent studies have shown that transgenic plants programmed to express dsRNAs corresponding to the most targeted region did not guarantee resistance against Tomato chlorotic mottle virus (ToCMoV) infections [50]. In addition, comparisons of viral DNA replication levels, viral mRNA levels and V-sRNA profiles did not identify a consistent correlation between different plants, although TYLCCNB was essential for the induction of characteristic disease symptoms and enhanced viral DNA accumulation in both *S. lycopersicum* and *N. benthamiana* plants [22]. In fact, similar scenarios have been observed in other virus-host systems. For example, in cassava-infecting begomoviruses, the abundance of V-sRNAs negatively correlated with viral titers in plants undergoing recovery from begomovirus infection [29]. By contrast, Cucurbit leaf crumble virus (CuLCrV) sRNA levels positively correlated with viral titers [29], [51]. One explanation for this discrepancy is that each virus-host combination might reflect specific characteristics; specifically a dynamic equilibrium or feedback loop during viral infection may affect sRNA levels in different model systems. Abundant viral DNA titers may give rise to more templates usable for viral mRNA transcription that may in turn result in aberrant templates for antiviral RDRs, reducing transcript levels of viral proteins. However, viruses have evolved to encode suppressors of RNA silencing to counter these defenses. Therefore, the accumulation levels of viral DNA depend ultimately on the rate of viral protein synthesis, viral RNA decay rates and possibly on the strength of the viral RNA silencing suppressor. Since *S. lycopersicum* is the natural host for TYLCCNV, it is likely that TYLCCNV has significantly adapted to this host and is able to combat host defense responses using its betasatellite.

## Materials and Methods

### Plant materials and viral inoculations

*S. lycopersicum* cv. Hongbaoshi and *N. benthamiana* plants at six-to-eight leaf stages were agroinoculated with infectious clones of the TYLCCNV isolate Y10 (pBinPLUS-Y10 1.7A) or in combination with its associated betasatellite (pBinPLUS-2β) or the *βC1*-mutant betasatellite (DNAβ-C1MB) at a 1∶1 ratio as described previously [22]. Mock-inoculations were performed by inoculating plants with *Agrobacterium tumefaciens* strain EHA105 containing pBinPLUS. Inoculated plants were kept in an insect-free chamber at 25°C with a 16∶8 h (light/dark) photoperiod. Systemically infected plant leaves were harvested 21 days post inoculation (dpi).

### Total DNA and RNA extractions

Total DNA from infected and mock-inoculated *S. lycopersicum* and *N. benthamiana* plant leaves was extracted using the CTAB method as described [52]. Total RNA of the systemic leaves (1 g) was extracted from pools of 8-10 systemically-infected or mock-inoculated *S. lycopersicum* or *N. benthamiana* plants using Trizol reagent (Invitrogen, Carlsbad, CA) as recommended by the manufacturer and then used for sRNA cloning and sRNA analyses.

### Enrichment of low molecular weight (LMW) RNA

The LMW RNA fraction was enriched using NaCl and PEG (molecular weight 8000) as described [53]. After denaturation with 1 volume of loading dye at 95°C for 3 min, isolated LMW RNAs were subjected to 15% denaturing polyacrylamide gel electrophoresis (PAGE) containing 8 M urea in 0.5X Tris-borate-EDTA buffer (TBE, 89 mM Tris, 89 mM borate, 2 mM EDTA) using synthetic RNA oligonucleotides of 18 and 28 nt in length as size markers and visualized under UV light following SYBR-Gold (Invitrogen) staining. sRNAs of 19–28 nt in length were excised from the gel, gel pieces crushed and incubated in 0.3 M NaCl overnight at 4°C allowing for nt diffusion out of the gel matrix. After centrifugation, supernatants were precipitated in ethanol and 1 µl of glycogen (20 mg/mL) (Roche, Mannheim, Germany) was used as a carrier. The pellet was washed with 80% ethanol and resuspended in nuclease-free ddH_2_O.

### sRNA library construction and sequencing

sRNA libraries were constructed as described [5]. Briefly, 18–28 nt sRNAs were sequentially ligated to a 3′ adapter and a 5′ accepter. After each ligation step, sRNAs were purified using 15% denaturing PAGE as described above. The final purified ligation products were reverse transcribed into cDNA using Superscript III reverse transcriptase (Invitrogen). The first strand cDNA was PCR amplified using T*aq* polymerase (Roche) and DNA amplicons from each library were purified and separately submitted for high-throughput sequencing using the Solexa platform (Illumina, San Diego, CA).

### Bioinformatic analyses of sRNA sequences

sRNA sequences were computationally analyzed by a set of Perl scripts from datasets generated from Illumina sequencing data. The adapter sequences were trimmed from raw reads and sRNAs between 16–27 nt in length were extracted. Only sRNA reads of sequences identical or complementary to TYLCCNV or TYLCCNB genomic sequences were recognized as V-sRNA or S-sRNA and those ranging between 20–24 nt in length were extracted for further analysis. The V-sRNA and S-sRNA reads were mapped to the TYLCCNV and TYLCCNB genome, respectively, using BLAST (http://www.ncbi.nlm.nih.gov/entrez). The data discussed in this publication have been deposited in NCBI's Gene Expression Omnibus and are accessible through GEO Series accession number GSE26368.

### Northern blot analysis of V-sRNAs

Approximately 40 µg of LMW RNA (prepared as described above) was subjected to 15% PAGE, transferred to an Hybond-N^+^ membrane (GE Healthcare, Buckinghamshire, UK) using a semi-dry transfer apparatus (Bio-Rad, Hercules, CA) and cross-linked using UV radiation. Equal loading of the gels and RNA integrity was determined by visualization of SYBR-gold-(Invitrogen) stained gels prior to blot transfer. The probe was prepared from *Bam*HI-digested full length TYLCCNV and labeled by random priming in the presence of [α-^32^P]dCTP. Blots were pre-hybridized and hybridized at 42°C overnight using ULTRAhyb®-Oligo hybridization buffer (Ambion, Austin, TX) as described [54]. Post-hybridization washes were performed twice using 2X SSC and 0.5% sodium dodecyl sulfate (SDS) at 42°C for 30 min. Hybridization signals were detected by exposing blots to autoradiographic film.

### Reverse Northern blot analysis of V-sRNA and S-sRNA

Six DNA fragments spanning the TYLCCNV genome and four that spanned the TYLCCNB genome were PCR-amplified using appropriate primer pairs (Table S1) using full length infectious clone pBinPLUS-Y10 1.7A or pBinPLUS-2β as templates [22]. The amplified PCR fragments were fractionated using a 1% w/v native agarose gel and transferred to Hybond N^+^ membrane (GE Healthcare) by capillary action using 20X SSC and cross-linked by UV illumination.

For preparation of the 21–24 nt RNAs, 60 µg LMW RNAs were subjected to 15% denaturing PAGE as described above. Approximately 1 µg of the purified 21–24 nt RNAs were dephosphorylated with alkaline phosphatase (CIP; New England Biolabs, MA) and labeled subsequently in a 30-µl reaction in the presence of [γ-^32^P]ATP and RNAsin (TaKaRa, Dalian, China) with 8U T4 polynucleotide kinase (New England Biolabs). Labeled 21–24 nt RNAs were used for reverse Northern blot hybridization using ULTRAhyb®-oligo hybridization buffer (Ambion) as described [54]. As a control, membranes were stripped and re-hybridized with labeled sRNAs extracted from mock-inoculated *N. benthamiana* and with probes derived from complete TYLCCNV or TYLCCNB sequences, respectively.

### Real-time quantification of viral RNA

Quantitative real-time PCR (qRT-PCR) was performed using a LightCycler® 480 Real-time PCR system (Roche). For relative quantification of viral RNA, 1 µg total RNA extracted from independent plant leaves was treated with DNaseI (Takara) and reverse transcribed following the manufacturer's instructions. In a 20 µl reaction, 1∶100 diluted cDNA was mixed with 1 µl of each 10 µM primer and 10 µl of 2X SYBR Green PCR supermix (Roche) following the manufacturer's recommendations. The primer pairs used for viral transcript quantification and internal standard GADPH are listed in Table S2. All PCR experiments were performed in triplicate and the results were analyzed using LightCycler® 480 software supplied by the manufacturer.

## Supporting Information

Table S1 **Primers used to amplify the TYLCCNV and TYLCCNB-derived fragments.**
(DOC)

Table S2 **Primers used for quantitative real-time PCR.**
(DOC)

Figure S1 **Polarity distribution of sequenced V-sRNA and S-sRNA.** Histograms represent the percent of (+) and (-) V-sRNAs (A) and S-sRNAs (B) of 21, 22 or 24 nt in length. Note that the values are calculated according to total reads of V-sRNAs or S-sRNAs. When calculated with unique sRNA sequences, approximately equal ratios of (+) and (-) V-sRNA and S-sRNA sequences are obtained (data not shown). Representation of Sl10A, Sl10A+10β, Nb10A, Nb10A+10β or Nb10A+10mβ was same as in Figure 1.
(TIF)

Figure S2 **The relative frequency of each V-sRNA or S-sRNA 5′-terminal nucleotide.** Histograms compare the 5′-termini of (+) and (-) V-sRNAs (A) and S-sRNAs (B) of 21, 22 and 24 nt in length, respectively. The frequency of each 5′-terminal nucleotide corresponding to V-sRNA or S-sRNA is calculated according to total reads of V-sRNAs and S-sRNAs. Representation of Sl10A, Sl10A+10β, Nb10A, Nb10A+10β or Nb10A+10mβ was same as in Figure 1.
(TIF)

Figure S3 **Hotspot profiles of V-sRNAs sequences captured from each library.** The 5′-ends of V-sRNAs sequences 21, 22 and 24 nt in length were plotted against the sense and antisense strands of the TYLCCNV genome, respectively. The values were calculated based on total reads of sequenced S-sRNAs. Note that the scale of the counts is different at both polarities. Representation of Sl10A, Sl10A+10β, Nb10A, Nb10A+10β or Nb10A+10mβ was same as in Figure 1.
(TIF)

Figure S4 **Hotspot profiles S-sRNAs captured from each library.** The 5′-ends of S-sRNAs sequences of 21, 22 and 24 nt in length were plotted against the sense and antisense strands of the TYLCCNB genome, respectively. The values were calculated based on total reads of sequenced S-sRNAs. Note that the scale of the counts is different at both polarities. Representation of Sl10A+10β, Nb10A+10β or Nb10A+10mβ was same as in Figure 1.
(TIF)
