# Supplementary material for: Characterization of Small Interfering RNAs Derived from the Geminivirus/Betasatellite Complex Using Deep Sequencing
Source: PLoS One. 2011 Feb 9;6(2):e16928. doi: 10.1371/journal.pone.0016928 (PMC3036729; doi:10.1371/journal.pone.0016928)
Supplement: Table S1 — Primers used to amplify the TYLCCNV and TYLCCNB-derived fragments. (DOC) [file pone.0016928.s001.doc]

| **Fragment** | **Primer** | **Primer sequence (5'-3')** | **Position** | **PCR fragment size (base pairs)** |
| --- | --- | --- | --- | --- |
| A1 | A1/F | ATGTGGGATCCTCTGCTCAAC | 128-148 | 408 |
|  | A1/R | AATATCATGACGAGCCTCATAG | 536-515 |  |
| A2 | A2/F | GCACATACTGGTAAAGTTATCTGTGT | 537-562 | 521 |
|  | A2/R | TTAATTCAACTGAGAATCATAAAAATAAA | 1058-1030 |  |
| A3 | A3/F | TAAATATTAAATTTTATATCATGATC | 1059-1084 | 545 |
|  | A3/R | ATGCGATCTTCGTCACCC | 1604-1587 |  |
| A4 | A4/F | TCTTTACCGCCCAGTTTTTAAGTG | 1605-1628 | 537 |
|  | A4/R | AGGGAAAAGGCCCCTAAAGAT | 2142-2122 |  |
| A5 | A5/F | CAATATATTGAGGGCCGAAGCT | 2143-2164 | 449 |
|  | A5/R | ATGCCTCCTCCTAATAAATTCAGAATAA | 2592-2565 |  |
| A6 | A6/F | TGACTGGTCAATTGGTGTCTCTC | 2593-2615 | 271 |
|  | A6/R | GCTTAACGTGAATACTTGGGC | 127- 107 |  |
| β1 | β1/F | CGACACGCGCGGCAGTGT | 1237-1254 | 319 |
|  | β1/R | ATACATATATATACGTATTCAAATATATG | 209-181 |  |
| β2 | β2/F | TCATACATCTGAATTTGTAAATACA | 210-234 | 357 |
|  | β2/R | ATGACTATCAAATACAACAACATGA | 566-542 |  |
| β3 | β3/F | GTTTATTTGTTGTGGATGATACATG | 567-591 | 335 |
|  | β3/R | CTTTTTTTTTTTCCGCTGCG | 901-881 |  |
| β4 | β4/F | GTAAATTTGAAACAAATAAAATCAACA | 902-928 | 335 |
|  | β4/R | CAAATTGATGACCGGAAGG | 1236-1218 |  |
| GUS | Gus/F | TCTAGATAATGTTCTGCGACGCTCAC |  | 320 |
|  | Gus/R | GGATCCGGCGAAATTCCATACCTGTTC |  |  |

**Table S1** Primers used to amplify the TYLCCNV and TYLCCNB-derived fragments.
