# Supplementary material for: Characterization of Small Interfering RNAs Derived from the Geminivirus/Betasatellite Complex Using Deep Sequencing
Source: PLoS One. 2011 Feb 9;6(2):e16928. doi: 10.1371/journal.pone.0016928 (PMC3036729; doi:10.1371/journal.pone.0016928)
Supplement: Table S2 — Primers used for quantitative real-time PCR. (DOC) [file pone.0016928.s002.doc]

**Table S2** Primers used for quantitative real-time PCR.

| Target | Primer | Primer sequence (5'-3') | Position | PCR fragment size (base pairs) |
| --- | --- | --- | --- | --- |
| IR | IR/QF | GGTGTCTCTCAAACTTGGCTAT | 2606-2627 | 165 bp |
|  | IR/QR | GGACCACTTTAAAAAAAATCGC | 33-12 |  |
| AV1 | AV1/QF | TTAGAGATCGTCGTCCTAGTGG | 706-727 | 168 bp |
|  | AV1/QR | GCTCCTTACAAGCATATTGTCC | 873-852 |  |
| AV2 | AV2/QF | CTCTCCTGATACATTAGGTTACGAT | 223-247 | 204 bp |
|  | AV2/QR | TGTTGGACCACATTTGTCTTC | 426-406 |  |
| AC1 | AC1/QF | ACCCACACTGTTCTCTTACGAA | 2519-2540 | 126 bp |
|  | AC1/QR | TATGCAGGTGAGGAGTCCCATC | 2436-2415 |  |
| AC2 | AC2/QF | ATGTTATTACGTCGTGGTTCC | 1318-1338 | 199 bp |
|  | AC2/QR | GTCGGGTTGACCTACCTTGTG | 1516-1496 |  |
| AC3 | AC3/QF | GCTAATCACGCCTAAACTATC | 1157-1177 | 183 bp |
|  | AC3/QR | AGGAACCACGACGTAATAACA | 1319-1339 |  |
| AC4 | AC4/QF | GTTGGCAGATTGGCAACCTCC | 2200-2220 | 155 bp |
|  | AC4/QR | CCCCAAACAGGTCAGCACATT | 2354-2334 |  |
| GADPH | GADPH/QF | GCAGTGAACGACCCATTTATCTC |  | 270 bp |
|  | GADPH/QR | AACCTTCTTGGCACCACCCT |  |  |
