# Supplementary figures and images for: Characterization of Small Interfering RNAs Derived from the Geminivirus/Betasatellite Complex Using Deep Sequencing
Source: PLoS One. 2011 Feb 9;6(2):e16928. doi: 10.1371/journal.pone.0016928 (PMC3036729; doi:10.1371/journal.pone.0016928)

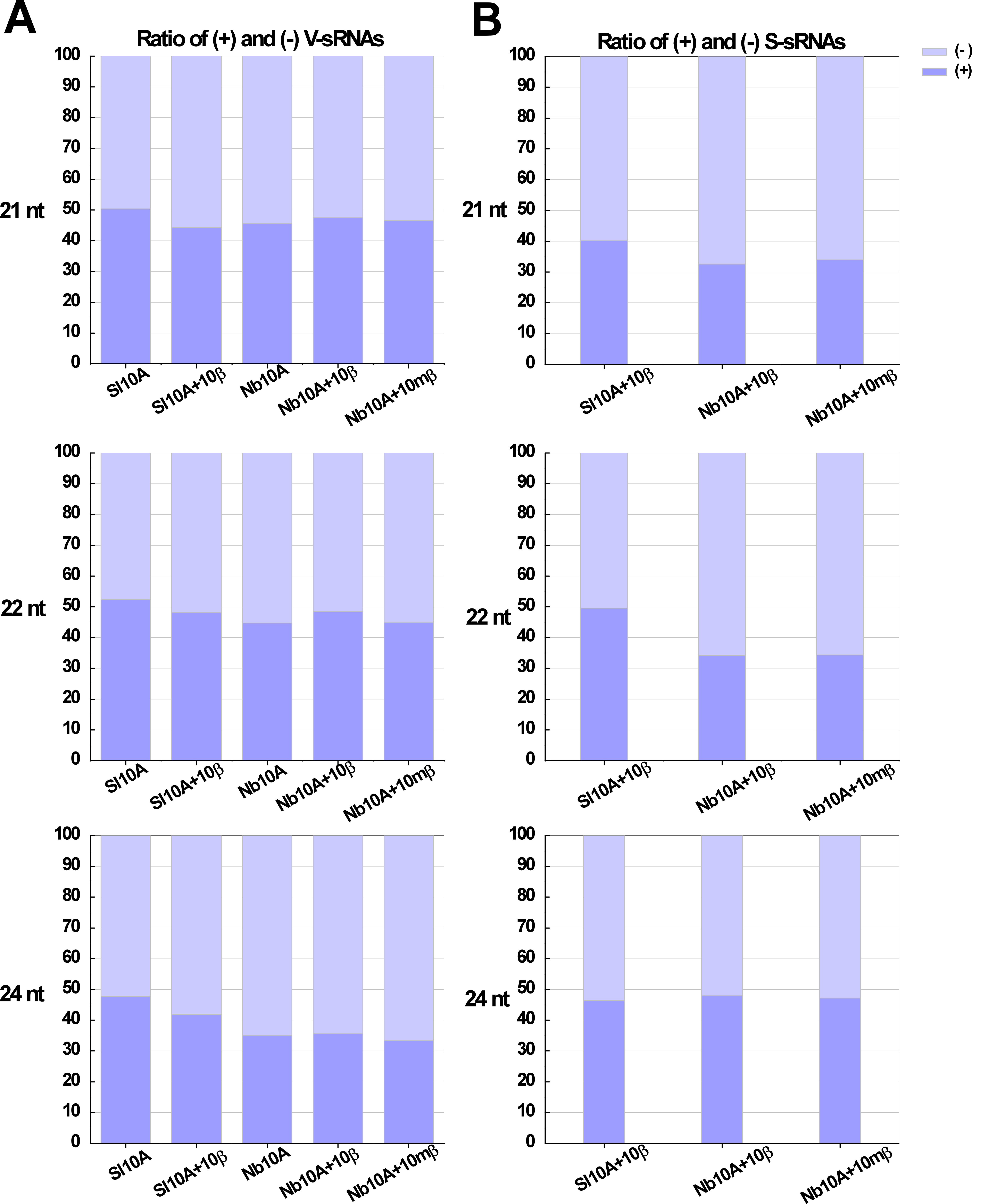

Supplement: Figure S1 — Polarity distribution of sequenced V-sRNA and S-sRNA. Histograms represent the percent of (+) and (-) V-sRNAs (A) and S-sRNAs (B) of 21, 22 or 24 nt in length. Note that the values are calculated according to total reads of V-sRNAs or S-sRNAs. When calculated with unique sRNA sequences, approximately equal ratios of (+) and (-) V-sRNA and S-sRNA sequences are obtained (data not shown). Representation of Sl10A, Sl10A+10β, Nb10A, Nb10A+10β or Nb10A+10mβ was same as in Figure 1. (TIF) [file pone.0016928.s003.tif]

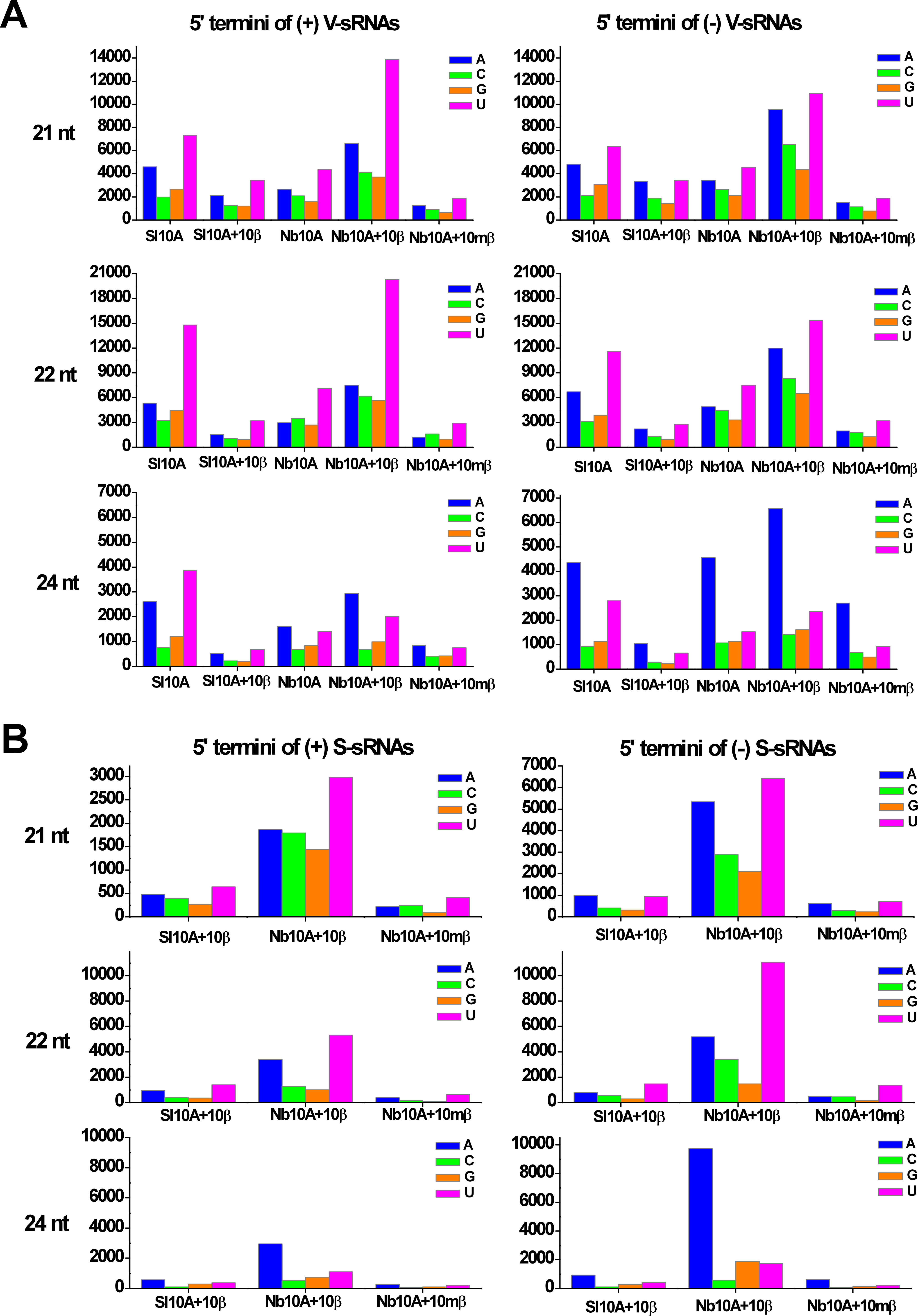

Supplement: Figure S2 — The relative frequency of each V-sRNA or S-sRNA 5′-terminal nucleotide. Histograms compare the 5′-termini of (+) and (-) V-sRNAs (A) and S-sRNAs (B) of 21, 22 and 24 nt in length, respectively. The frequency of each 5′-terminal nucleotide corresponding to V-sRNA or S-sRNA is calculated according to total reads of V-sRNAs and S-sRNAs. Representation of Sl10A, Sl10A+10β, Nb10A, Nb10A+10β or Nb10A+10mβ was same as in Figure 1. (TIF) [file pone.0016928.s004.tif]

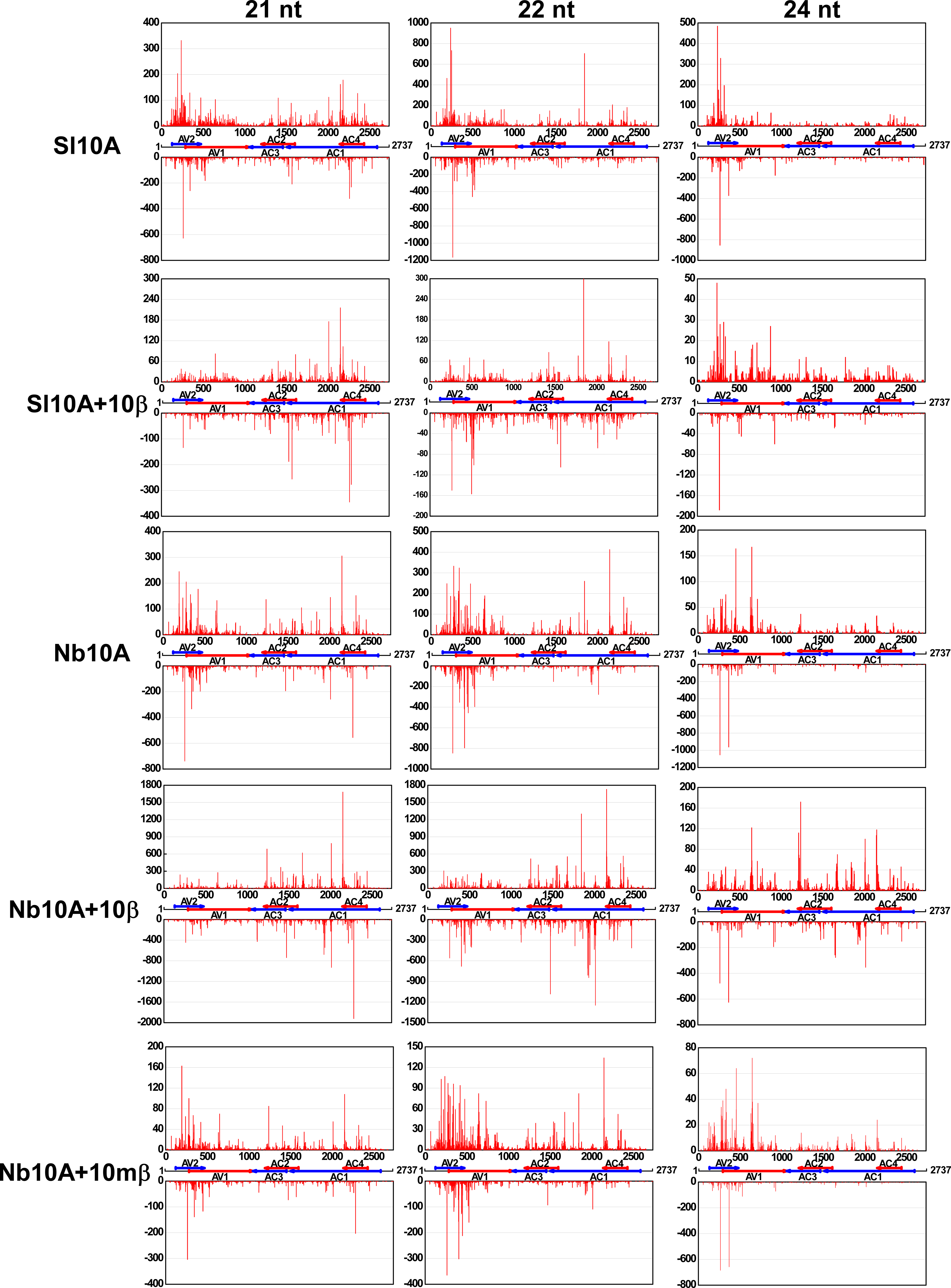

Supplement: Figure S3 — Hotspot profiles of V-sRNAs sequences captured from each library. The 5′-ends of V-sRNAs sequences 21, 22 and 24 nt in length were plotted against the sense and antisense strands of the TYLCCNV genome, respectively. The values were calculated based on total reads of sequenced S-sRNAs. Note that the scale of the counts is different at both polarities. Representation of Sl10A, Sl10A+10β, Nb10A, Nb10A+10β or Nb10A+10mβ was same as in Figure 1. (TIF) [file pone.0016928.s005.tif]

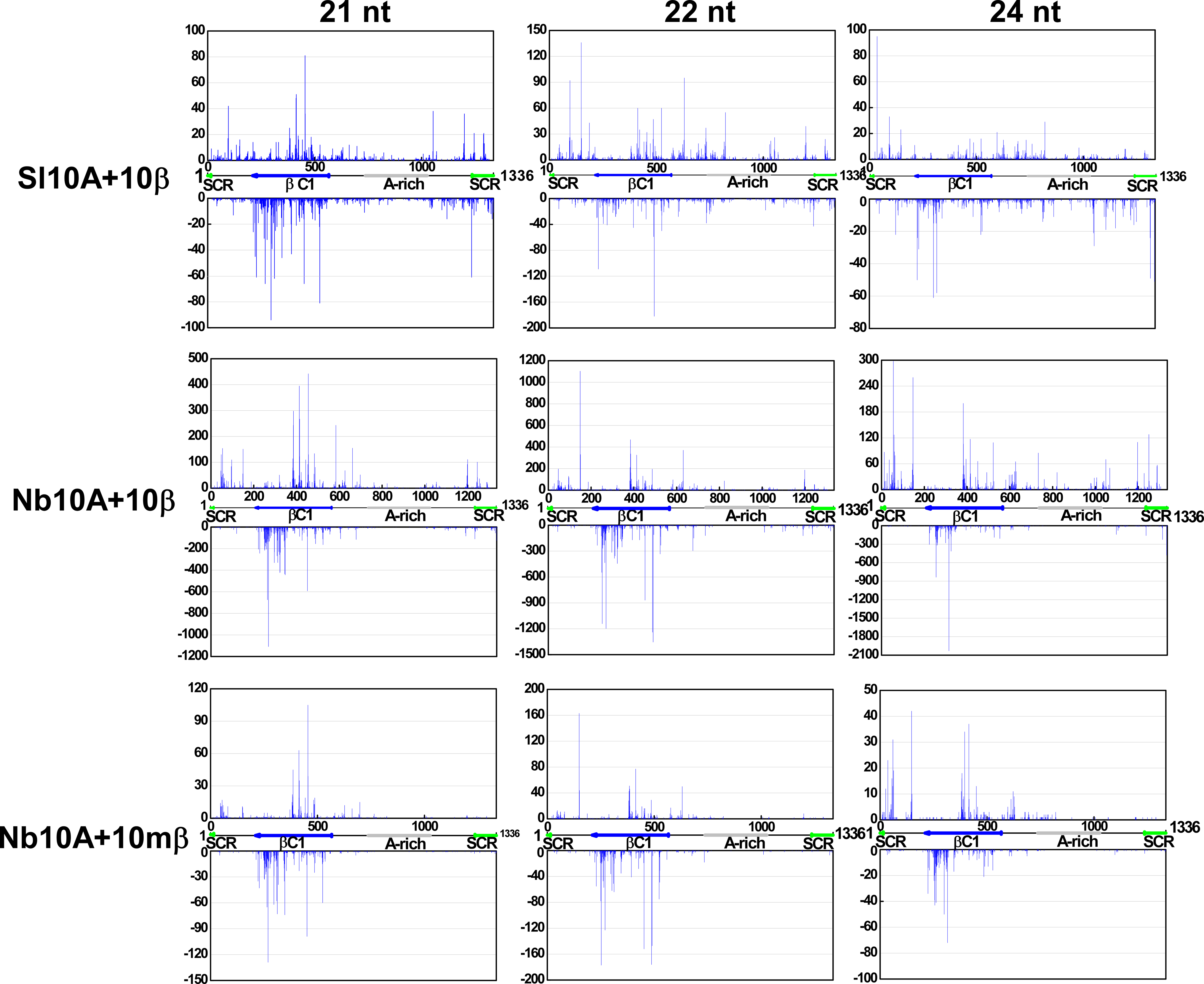

Supplement: Figure S4 — Hotspot profiles S-sRNAs captured from each library. The 5′-ends of S-sRNAs sequences of 21, 22 and 24 nt in length were plotted against the sense and antisense strands of the TYLCCNB genome, respectively. The values were calculated based on total reads of sequenced S-sRNAs. Note that the scale of the counts is different at both polarities. Representation of Sl10A+10β, Nb10A+10β or Nb10A+10mβ was same as in Figure 1. (TIF) [file pone.0016928.s006.tif]
